# Programming Quantum Neural Networks on NISQ Systems: An Overview of Technologies and Methodologies

**DOI:** 10.3390/e25040694

**Published:** 2023-04-20

**Authors:** Stefano Markidis

**Affiliations:** KTH Royal Institute of Technology, 114 28 Stockholm, Sweden; markidis@kth.se

**Keywords:** Quantum Neural Networks, QNN programming frameworks, Amazon Braket, D-Wave Ocean, Intel HQCL, Microsoft QDK, Nvidia CUDA Quantum, OriginQ QPanda, Qiskit Machine Leaning, PennyLane, Rigetti Grove, Strawberry Fields, TensorFlow Quantum, Torch Quantum, Zapata Orquestra

## Abstract

Noisy Intermediate-Scale Quantum (NISQ) systems and associated programming interfaces make it possible to explore and investigate the design and development of quantum computing techniques for Machine Learning (ML) applications. Among the most recent quantum ML approaches, Quantum Neural Networks (QNN) emerged as an important tool for data analysis. With the QNN advent, higher-level programming interfaces for QNN have been developed. In this paper, we survey the current state-of-the-art high-level programming approaches for QNN development. We discuss target architectures, critical QNN algorithmic components, such as the hybrid workflow of Quantum Annealers and Parametrized Quantum Circuits, QNN architectures, optimizers, gradient calculations, and applications. Finally, we overview the existing programming QNN frameworks, their software architecture, and associated quantum simulators.

## 1. Introduction

Quantum computing is emerging as a disruptive and promising approach to attacking computational and data analysis problems. Quantum computing relies on three essential quantum effects inaccessible directly by classical computing systems [1,2]: (i) calculation on a superposition of quantum states somehow reminiscent of parallel computing, (ii) entanglement to correlate different quantum states, and (iii) quantum tunneling. These three effects can be used to seek the so-called *quantum advantage* [3] over classical algorithms by, for instance, computing in a superposition or hopping between optimization landscapes via quantum tunneling. The first critical quantum computing applications with quantum advantage are in the area of cryptology and search algorithms with the most famous Shor’s and Grover’s algorithms. Today, researchers’ attention started focusing on the possibility of developing quantum Machine Learning (ML) applications [4,5] for classical and *quantum data*, e.g., data encoded as a superposition of quantum states, resulting from quantum simulations or sensing.

The early quantum ML approaches rely on the so-called quantum Basic Linear Algebra Subprograms (qBLAS) primitives [4]. Examples of qBLAS routines are the Quantum Fourier Transform (QFT), Quantum Phase Estimation (QPE) for obtaining eigenstates and eigenphases, and the Harrow–Hassidim–Lloyd (HHL) algorithm for solving linear systems [6]. These qBLAS-based ML methods consist of classical ML approaches, such as the quantum Principal Component Analysis (PCA) [7], quantum regression with least-square fitting [8], quantum topological analysis [9], quantum Bayesian inference [10], and quantum Support Vector Machine (SVD) [11]. While these quantum ML methods exhibit a clear quantum advantage concerning corresponding classical algorithms, severe constraints, such as embedding classical data into quantum states, the need for quantum memory, qRAM [12], and output analysis and post-processing, limit their immediate applicability on Noisy Intermediate-Scale Quantum (NISQ) computers [13].

Conversely, a second family of quantum ML methods, based on heuristics and hybrid classical-quantum computing instead of purely quantum BLAS primitives, can readily exploit the NISQ systems, albeit not demonstrating a crystal clear quantum advantage yet [14,15]. These methods target the development of the so-called Quantum Neural Networks (QNN). Similarly to classical Neural Networks (NN), in QNNs, an optimization process provides the weights and biases of a neural network by minimizing a loss function measured (or sampled) on the quantum computers. This survey focuses on this second family of quantum ML methods that can readily use NISQ systems.

With quantum computers hardware becoming widely available on several cloud services (e.g., via IBM, Google, Rigetti, Amazon Braket, and Microsoft Quantum Azure clouds, to mention a few examples), there is an increased interest on the software quantum computing side, specifically in designing and developing programming abstractions, patterns, and templates to assist application developers and data scientists in implementing QNNs in a productive and high-performance manner.

Regarding software development for quantum computing, there is already an ecosystem of programming approaches to express quantum algorithms in terms of the quantum gate and circuit abstractions. Examples of established programming systems [16] for the quantum computing models are the QASM [17], akin to the assembly language for classical CPUs, IBM’s Qiskit [18], Google’s Cirq and Rigetti’s PyQuil [19], to mention a few. These programming models use an offloading paradigm, similar to the one used for Graphical Processing Units (GPU) programming languages: the quantum language provides means to define quantum circuits on a CPU, *offload* or *launch* the quantum circuit on the QPU from the CPU (via a connection to the cloud), execute the circuit, measure an observable several times, and finally return the measurements to the CPU.

While these programming systems enable the development and implementation of quantum computing primitives, such as QFT and QPE, data scientists and application developers require higher-level programming models that allow them to express their algorithms in terms of quantum neural units, layers, loss functions, optimizers, and automatic differentiation (to cite a few of the technologies critical to QNN development). Higher-level programming frameworks, such as TensorFlow [20] or PyTorch [21] for quantum computers, are needed to increase the programmer’s productivity in developing applications on quantum computers. In addition, together with means to express neural network concepts and abstractions for training QNNs, quantum programming frameworks must integrate with classical deep-learning frameworks to leverage existing software infrastructure.

In the last years, the number of QNN software has bloomed, leading to the transition of classical NN software to quantum-enabled versions (examples are TensorFlow Quantum and Torch Quantum), development of QNN abstractions and templates on top of existing quantum computing frameworks (for instance, the Qiskit machine learning library built on the top of IBM Qiskit) and creation of new programming frameworks, such as the Xanadu’s PennyLane, targeting specifically differentiable programming and QNNs.

This article aims to provide an outlook on the different technologies and methodologies used for developing QNNs, and an overview of existing higher-level QNN programming frameworks. Section 2 reviews the current target quantum computer architectures, approaches for implementing QNNs, and methodologies, including QNN approaches, optimizers, differentiation techniques, and applications. In Section 3, we overview different and emerging software frameworks for developing QNNs, emphasizing characteristic features, software organization, and associated computer simulators. Finally, we summarize the review and outline future challenges for QNN frameworks in Section 4.

## 2. Quantum Neural Network Technologies and Methodologies

This section provides an overview of the target quantum architectures on which QNNs can be deployed, the methods and algorithms for implementing QNN, and essential technologies in use, such as QNN building blocks, optimizers, and automatic differentiation techniques.

### 2.1. Target NISQ Architectures for QNN

At a high level, we can divide the QNN target quantum computer architectures into two broad categories:

1. **Quantum Annealers (QA).** In this quantum computing approach, the loss function is expressed as the cost function of a QUBO (Quadratic Unconstrained Binary Optimization) problem, equivalent to the Hamiltonian of an Ising system [22]. Currently, the most established QA machines are from the Canadian D-Wave. Additional companies working on and researching the development of QA platforms are Fujitsu, with its Digital Annealer [23,24], Toshiba, with its Simulated Bifurcation Machine (SBM) [25], NEC (developing a QA processor using the so-called Lechner-Hauke-Zoller architecture [26]), and Qilimanjaro Quantum Tech, a spinoff of the Barcelona Supercomputing Center.
2. **Universal Gate Quantum Computers.** In this quantum computing model, the QNN loss function is expressed in terms of a measurement associated with a parametrized quantum circuit using universal quantum gates. Differently from QAs, universal quantum computers can solve problems beyond optimization tasks, formulated as the minimization of an Ising Hamiltonian.
  There are two formulations for the universal quantum gates that can be used to express the QNN loss function:
  (a) **Discrete Qubit-Based Quantum Computing.** Qubit-based architectures are the most established general-purpose quantum computing approach. They use the discrete formulation of a quantum state equivalent to a bit [27]. The qubit |ϕ〉 is expressed as the combination (or a superposition) of the states |0〉 and |1〉 as $|\phi \rangle =\phi_{0}|0\rangle +\phi_{1}|1\rangle$. We use a set of discrete complex-valued coefficients, such as $\phi_{0}$ and $\phi_{1}$, whose modulus squared corresponds to the probability of measuring |0〉 and |1〉 in the qubit system measurement.
    Discrete-qubit QNNs rely on parametrizing discrete quantum gates, such as rotation and Pauli gates. Discrete qubit-based QNNs are generally considered a good match for classification tasks because of the discrete nature of the problem. Among the most famous hardware implementations (and associated software) in this category, there are IBM (Qiskit), Google (Cirq), Rigetti (Forest), and OriginQ (Qpanda) quantum computers. All these implementations use superconducting/transmon qubit technologies. Another prominent company is Pasqal, with a neutral atom quantum computer that can be used in analog and digital versions [28].
  (b) **Continuous Variable (CV) Quantum Computing.** The CV quantum computing approach is the analog version of quantum computing [29], still using a QC gate formulation [30]. CV is based on the concept of *qumode*, the continuous analogous of the qubit.
    The qumode |ψ〉 is expressed in the basis expansion of quantum states, as |ψ〉=∫ψ(x)|x〉, where *x* are the real-valued eigenvalues and |x〉 are the eigenstates of the $\hat{x}$ quadrature, $\hat{x}|x\rangle =x|x\rangle$. CV quantum computing and CV QNN use continuous quantum gates, such as displacement, squeeze, rotation, and Kerr gates, to express the quantum circuit operations. Because of the continuous approach, CV QPC is regarded as an excellent fit for QNN regression-like tasks. In addition, CV QNNs are a critical building block for developing quantum Physics Informed Neural Networks (PINN) using CV gates [31].
    The most established technology to implement CV quantum gates is photonics. The Canadian Xanadu is among the most active and established companies developing photonics quantum chips. Among others, Xanadu is one of the leading companies for the development of QNN programming frameworks: Strawberry Fields (and, most importantly, its integration with a TensorFlow backend) and PennyLane are important examples of programming frameworks that allow for CV QNNs.

### 2.2. Quantum Neural Network Input Data

QNNs can operate on two kinds of data:

1. **Classical Data.** In this case, the training datasets consist of classical data, such as the pixel values of an image. When QNN uses classical data, then an encoding of the classical data into quantum states is required. The most used encoding techniques are amplitude, angle, basis, and Hamiltonian encodings [5,32]. The encoding often requires the usage of an additional QNN layer.
2. **Quantum Data and Integration with Quantum Simulators.** Quantum data are encoded as a superposition of quantum states, where each quantum state has an associated amplitude and a phase. Quantum data cannot be generated classically but might result from quantum sensing or quantum circuit running a quantum algorithm or quantum simulations. An example of code using quantum data is the TensorFlow Quantum Hello Many-Worlds code [33] (https://github.com/tensorflow/quantum/blob/research/binary_classifier/binary_classifier.ipynb, accessed on 3 April 2023) that classifies two classes of quantum data points distributed in the Bloch sphere [27].
  Classical NN cannot operate on quantum data, and QNN provides the only mean to process quantum data directly. If the QNN uses quantum data, then a special data loader or integration with quantum simulations programming frameworks, such as OpenFermion [34], and PySCF [35] are required. All the main QNN frameworks provide integration of quantum simulations as part of the same package or integration with OpenFermion and PySCF.

### 2.3. Quantum Neural Network Approaches

This section discusses the two main algorithmic strategies for developing QNN on QAs and universal gate-based quantum computers.

#### 2.3.1. QNN with Quantum Annealers

Historically, the first approach to tackling QNN development relies on using QAs, specialized quantum computers, on solving optimization problems [36,37]. In essence, QAs provide the ground state of a Hamiltonian of an Ising system (used, for instance, in magnetism problems and energy-based ML methods). If we formulate the QNN loss function as an Ising model, then finding the quantum system ground state corresponds to finding the loss function minimum. In the case of QA-based QNNs, the loss function can be expressed as:

(1) $$L=\Sigma_{i}h_{i}s_{i}+\Sigma_{i,j}J_{i,j}s_{i}s_{j},$$

where $J_{i,j}$ are the QNN weights, $h_{i}$, the QNN biases, and $s_{i}$ the spins (encoded in the qubit) that can take only the values +1 and −1. The QAs minimize the loss function of Equation (1), returning the weights and biases. To run on the quantum computer, Equation (1) must be first formulated in an equivalent QUBO matrix format: $L=X^{T}QX$ with $x_{i}=\left(1-s_{i}\right)/2$ (the so-called *spin to binary relation*). Then, the loss function must be mapped to the underlying QA hardware and network topology through a process called *graph embedding* [38,39]. In the case of D-Wave systems, the embedding is into a Chimera graph.

**Figure 1:**
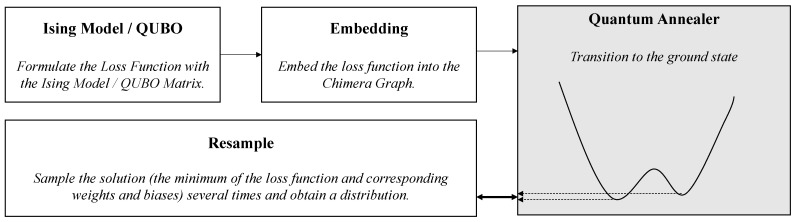
Diagram of the basic workflow for training a QA-based QNN.

The workflow to run a QNN on QAs is represented in Figure 1. The QNN loss function is first formulated as a QUBO problem and then embedded into the underlying quantum computer topology graph. These steps are performed on the classical computer. The QAs calculate the loss function minimum (equivalent to the ground energy state of Ising Hamiltonian) and associated QNN weights and biases. A resampling phase allows for loss function minimum sampling several times. Because QA-based QNNs use Ising Hamiltonian in their formulation, they can straightforwardly represent energy-based NNs [40], such as Hopfield networks [41], Boltzmann machines [42], Restricted Boltzmann Machines (RBM) [43], and used as a part of the Deep Belief Network (DBN) model [44].

#### 2.3.2. QNN with Parametrized Quantum Circuits

The second QNN class can use universal quantum computers instead of QAs and goes under the name of Parametrized Quantum Circuit (PQC) [45], or Variational Quantum Circuits (VQC) [46,47]. The basic fundamental PQC idea is to express the weights and biases of the neural network as parameters of an exemplar quantum circuit (also called the *Ansatz*) and adapt the parameters to minimize a loss or cost function using a classical optimizer, such as Stochastic Gradient Descent (SGD) [48] or Adam [49] optimizers.

**Figure 2:**
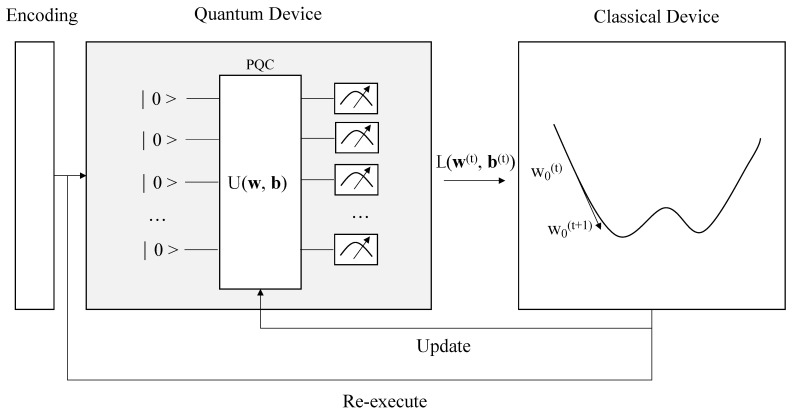
Diagram of the basic workflow for training a PQC-based QNN.

Figure 2 shows the typical workflow when running a PQC. The first step randomly initializes the QNN weights *w* and biases *b*. These are parameters characterizing a gate in the PQC. For instance, the angle of a rotation gate can be a QNN parameter, e.g., a QNN weight. Then for each training sample, we first encode the input data (an image, for instance) into a quantum state using an encoding layer; we then execute the measure of the PQC results with current *w* and *b* (this corresponds to apply a unitary circuit U(w,b) to the encoded sample |0〉 as in U(w,b)|0〉=|ψ(w,b)〉). The norm of the difference between the measurement and training sample label will provide the loss function. For instance, a loss function is calculated using the PQC measurement and label data ($y_{|0\rangle }$):

(2) $$L=∥\langle y|U\left(w,b\right)|0\rangle -y_{|0\rangle }∥.$$

Finally, similarly to NN, we can use the back-propagation step to update the QNN parameters. The loss function value drives an optimization step to determine new updated parameter values (*w* and *b*) to minimize the loss function. We repeat this process for each training sample. An essential point about PQC loss functions is that they are not limited to QUBO problems such as QA but are more general. In fact, it is possible to solve Ising problems using PQC.

QNNs, implemented with QPC, are a very active and fast-growing research area. Several QNNs architectures, often mimicking the classical counterparts, have developed, including quantum fully connected, convolutional [30,50]/quanvolutional [51], recurrent [30], GAN [52], and tensor networks [53].

A significant research effort is made to address the so-called *barren plateau* problem [54] for the QPC optimization landscape: in several PQCs, the average value of the gradient tends to zero, and as the Hilbert dimension increases, the more states will lead to a flat optimization landscape. For this reason, the optimizer cannot converge to the minimum of the loss function. To address this issue, a few techniques are proposed, including an initialization technique to initialize randomly only a subset of the parameters [55], using a local instead of a global loss function [56], and data re-uploading [57].

### 2.4. Quantum Neural Network Architectures

In the case of PQC, it is possible to build QNNs by combining different layers in a similar way to the classical NN. The most common kinds of QNN layers are:

- **Encoding/Embedding Layers.** These layers are used to encode classical data into quantum Hilbert space. Basically, the encoding process is equivalent to a **feature map** that assigns data to quantum states [58,59]. Inner products of such data-encoding quantum states give rise to **quantum kernels**. These feature maps are used in QNNs as a way to perform nonlinear transformations, akin to activation functions in NN, on the input data.
  Common feature maps used in the QNNs are amplitude, angle, basis, and Hamiltonian encodings. Amplitude and angle encodings map classical data to the amplitudes and phases of a quantum state, respectively. Basis embedding encodes the binary feature vector into a basis state. Hamiltonian encoding associates a system’s Hamiltonian with a matrix representing the data transformation. An example of Hamiltonian encoding is using a quantum circuit with single-qubit rotations to encode the input data. This encoding using multiple quantum rotation gates, for instance, allows us to express quantum models as Fourier-type sums [60]. In CV QNNs, the most used encoding is displacement embedding, which encodes features into the displacement of qumodes amplitudes or phases.
  Encoding layers are critical for developing QNN as the data-encoding strategy largely defines the QNN expressivity, e.g., the features QNN can represent [59,61]. Feature maps are critical building blocks for developing scientific quantum machine learning and Differentiable Quantum Circuit (DQC) [62,63,64].
- **Variational Layers.** These layers are the PQC building block and include trainable parameters (*w* and *b*) in the quantum circuit. These parameters are optimized during the QNN training. They typically consist of a series of single- and two-qubit gates, with associated gate parameters optimized during training.
- **Entangling Layers.** An important subclass of variational layers is the entangling layers class that creates entangled quantum states. These layers comprise one-parameter single-qubit rotations on each qubit, followed by a CNOT gate chain. *Basic entangling layers* have a CNOT gate chain connecting every qubit with its neighbor. *Strongly entangling layers* feature a CNOT gate chain also connecting non-neighbor qubits [65]. *Random entangling layers* have single qubit rotations and CNOT gates, acting on randomly chosen qubits. Another entangling layer is the so-called *2-design*, consisting of qubit rotations and Controlled-Z (CZ gate) entangling layers [56].
- **Pooling Layers.** Pooling layers reduce the quantum circuit size by typically grouping together several qubits and performing operations that reduce the quantum state dimensionality. The way to implement pooling layers is to measure a qubit subset of the qubits and then use the measurement to control the following operations. Pooling layers are an important component of quantum convolutional networks [66].
- **Measurement Layers.** Measurement layers are used to measure classical information (bit) from the superposition of quantum states in the QNN. Measurements layers typically are single-qubit measurements of the output qubits that provide classical values for the QNN output.

**Figure 3:**
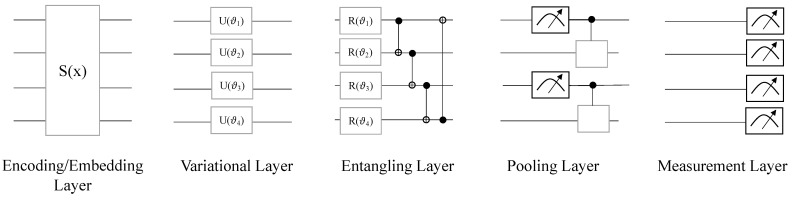
Examples of common quantum layers used for constructing QNNs: an encoding/embedding layer using a circuit block S(x) as Hamiltonian encoding, a variational layer with a unitary gate *U* with four parameters ($\theta_{1}$, $\theta_{2}$, $\theta_{3}$ and $\theta_{4}$), a simple entangling layer with rotation operation (*R*) and CNOT gates operating on neighbor qubits, a pooling layer used for quantum convolutional networks, and finally a measurement layer.

In addition, the basic *CV QNN layer* consists of displacement, squeezing gates, interferometers to mimic the linear transformation of a neural network, and a Kerr gate to introduce nonlinearity to mimic the neural network activation function [30]. Figure 3 shows a few simple QNN examples used to construct the full PQC.

How to compose QNN layers automatically into PQC for solving a specific problem and minimizing the noise impact on real quantum machines is an active research area and led to the development of the SuperCircuit [67] and Supernet [68].

### 2.5. Optimizers for Parametrized Quantum Circuits

A key technology for training the PQC is the optimizer that allows us to find the minimum or maximum of a multi-variable function, e.g., the loss function in our case. The optimizers can be divided into two broad categories:

- **Gradient-free Optimizers.** Gradient-free optimization methods are techniques that do not require the calculation of the gradient for the back-propagation step [69], reducing the complexity of performing differentiation on a quantum circuit. For this reason, they were widely used in developing the first QNNs. This optimizer class includes the Nelder–Mead [70] and COBYLA algorithms [71]. These gradient-free optimizer methods are often provided within the QNN frameworks (e.g., they are readily available in Qiskit) or available via external packages, such as SciPy [72].
- **Gradient-based Optimizers.** Gradient-based optimizers require gradient calculation on the QNN. Compared to gradient-free optimizers, gradient-based optimizers provide advantages from convergence guarantees [73] and are the method of choice in modern QNNs. Examples of gradient-based optimizers are the deep-learning workhorse algorithms, such as the Stochastic Gradient Descent (SGD) and Adam. These optimizers are readily available in many QNN frameworks or are obtained from integrating QNN programming frameworks with TensorFlow/Keras and PyTorch. For instance, Quantum TensorFlow and Strawberry Fields can readily use TensorFlow 2 and Keras optimizers.
  Together with traditional ML optimizers, additional optimizers are used to reduce evaluation costs and address the problem of the *barren plateau*. For instance, a popular optimizer, robust to noise, is the Simultaneous Perturbation Stochastic Approximation (SPSA) [74], which is a stochastic method to approximate the loss function gradient. In this optimizer, the loss function is evaluated using perturbed parameter vectors: each component of the parameter vector is shifted by a random value. Another example is the doubly stochastic gradient descent method [73] that reduces the cost of evaluating the gradient at each iteration by evaluating only a random subset of the gradient components. Additionally, the Quantum Natural Gradient (QNGOptimizer) [75,76] improves the quality of our optimization landscape (affected by the *barren plateau* problem) by moving along the steepest direction in the Hilbert space instead of the parameter space.

### 2.6. Differentiation for Parametrized Quantum Circuits

When using classical gradient-based optimizers, the optimization step relies on calculating the gradients of the loss function in the optimization landscape. In classical NN, derivatives on the neural network are calculated using the automatic differentiation technique [77]. The fact that the loss function is defined as a quantum circuit constitutes a challenge for this formulation. Some differentiation approaches [78,79] for PQC on quantum hardware and simulators are possible:

- **Parameter Shift Rule/Quantum Automatic Differentiation**. This differentiation technique allows calculating derivatives using the same PQC with a difference only in a shift of the argument [80,81]. The basic idea of this technique is to consider these quantum functions as Fourier series. The partial derivative of a function can then be formulated as a linear combination of them. An intuitive example of the parameter shift rule workings (https://pennylane.ai/qml/glossary/parameter_shift.html, accessed on 3 April 2023) is the calculation of sin(x) that is equivalent to a *shifted* formulation: 1/2sin(x+π/2)−1/2sin(x−π/2). The same underlying algorithm can be reused to compute both sin(x) and its derivative at ±π/2. This works for many PQCs of interest, and the same PQC can be used to evaluate both the loss function and its gradient on a quantum computer.
- **Numerical Derivative.** Numerical derivative methods are based on finite-different discretization. This differentiation calculation can run on a quantum computer as a *black box* as it requires PQC evaluations common at two separated points in the parameter *w* at a distance Δ: $f^{\prime }\left(w\right)=\left(f\left(w+\Delta \right)-f\left(w\right)\right)/\Delta$ in a simple case of forward finite-difference. The challenge with this technique is the number of PQC evaluations that this method requires and the accuracy (given the dependency on Δ).
- **Adjoint Derivative (for quantum simulators)**. This differentiation method applies only to quantum computer simulators, as the method requires examining and modifying the full quantum state vector. This method works iteratively by applying the inverse (adjoint) gate [82] and has significantly lower memory usage and a similar runtime than the *backprop*. For this reason, this is the method of choice for HPC implementation of automatic differentiation on quantum computer simulators.
- **Quantum analytic descent (on classical computers)**. This method constructs a classical model approximating the optimization landscape in the minimum proximity by using a sum of multilinear trigonometric terms in each parameter so that the gradients can be easily calculated on a classical computer that is computationally convenient [83].

### 2.7. Applications

QNNs have been used in many applications similarly to classical NN. QAs and D-Wave machines are among the most successful quantum computing platforms in existing QML applications. Few examples include image classification (MNIST dataset) [44,84], computational biology [85], and high-energy physics [86]. The PennyLane QNN framework has found applications in image classification [87], cyber-security [88], medical [89], and high-energy physics problems [90,91]. TensorFlow Quantum has been used for image classification [66], remote sensing [92], and medical applications [93].

## 3. Quantum Neural Network Software Frameworks

This section briefly reviews existing and emerging QNN programming frameworks. We note that new programming environments are continuously developed as new approaches and quantum computer systems arise. The list we present strives to cover the most used programming approaches, but it is necessarily not exhaustive.

### 3.1. Amazon Braket SDK

Amazon offers its quantum cloud, called Amazon Braket. Unlike many other vendors, Amazon does not develop quantum hardware; instead, it provides services over third-party quantum hardware [94] using superconducting, trapped ion, neutral-atom, and photonics technologies. Current quantum hardware providers within Amazon Braket include IonQ, Oxford Quantum Circuits (OQC), QuEra, Rigetti, and Xanadu.

QNNs can be programmed using the Amazon Braket Python SDK that provides means of connecting quantum computers and simulators and the basic programming abstractions for PQC programming. While Amazon Braket SDK does not offer a dedicated library for QNNs, it is possible to develop a PQC from scratch using Braket gates and measurement features (https://aws.amazon.com/blogs/quantum-computing/aioi-using-quantum-machine-learning-with-amazon-braket-to-create-a-binary-classifier/, accessed on 3 April 2023). Braket does not provide optimizers; however, it is possible to use the SciPy optimizers, such as the second-order L-BFGS [95]. Amazon Braket also provides a set of local and on-demand quantum computer simulators. The on-demand simulators can use distributed HPC systems and execute elastic Amazon Web Services (AWS) runs. Braket SDK simulators include state-vector, density matrix, and tensor-networks simulators. An important aspect of Amazon Braket is that it provides access to several other QNN programming frameworks, such as PennyLane and Qiskit.

### 3.2. D-Wave Ocean

D-Wave provides a software framework called Ocean SDK to connect and run quantum optimization problems on the D-Wave QA machines. As mentioned previously, QAs must first have the problem cast to a QUBO formulation and then embedded into the underlying qubit topology (a Chimera graph in the case of the D-Wave machines). To convert the Ising problem to a QUBO problem, the pyQUBO library [96] is typically used. The method EmbeddingComposite embeds the QUBO to the Chimera graph of the physical QA in D-Wave. After the problem is embedded in the QUBO form, it can be run calling the method sample_qubo(...,num_sample=...) providing the number of samples. Different samplers are provided in D-wave are provided: quantum, hybrid, and classical solvers, including *simulated annealing*, *tabu* (a heuristic that employs local search), among the others. At high-level, the D-Wave Ocean framework consists of these different software components:

- **Problem Definition.** This software layer provides tools for defining optimization problems that can be solved using quantum annealing. It includes tools for defining variables, constraints, and objective functions.
- **Samplers.** The Ocean sampler allows us to access different compute resource (CPU/GPU/QPU) and different optimization techniques.
- **Embedding.** This software layer provides tools for mapping high-level problem definitions onto the hardware constraints defined by the sampler. Using a QA, Ocean allows us to map the problem defined in the problem definition phase onto the hardware qubits of the QA.
- **Utilities.** This component provides a set of utility functions that can be used to analyze the results of the quantum annealing runs, visualize the embeddings, and debug the models.

OpenJIJ (https://github.com/OpenJij/OpenJij, accessed on 3 April 2023) is an open-source library that simulates the QAs and can be used to experiment without the D-Wave computers.

### 3.3. Intel HQCL

Intel has developed a Software Development Kit (SDK) called Intel Quantum SDK [97]. Currently, the Intel Quantum SDK supports only PQC simulations; however, it is expected to support real quantum hardware in the future. In particular, Intel is investing in quantum dot-based quantum computers. Future Intel Quantum SDK releases will include a quantum dot qubit simulator and an Intel quantum dot qubit device. The Intel Quantum SDK allows writing PQC based on C++ and an LLVM-based compiler toolchain that optimizes the quantum runtime for executing hybrid quantum-classical workloads [98]. The Intel quantum computer simulator is called IQS, for Intel Quantum Simulator.

Regarding PQC implementations, Intel provides the Hybrid Quantum-Classical Library (HQCL), a high-level library to express Hybrid Quantum-Classical algorithms exploiting Intel Quantum SDK and run on the quantum computer simulator [99].

### 3.4. Microsoft Azure QDK

Microsoft Azure Quantum provides access to quantum computers from several vendors, including IonQ (trapped-ion Technology), Honeywell (trapped-ion technology), Quantum Circuits Inc. (superconducting qubit technology), Rigetti (superconducting qubit technology), and Pasqal (neutral atom technology). Microsoft Azure Quantum allows for submitting provider-specific formatted quantum circuits (for instance, in QASM or JSON format) to supported quantum computing targets via the Azure Quantum services.

Microsoft also provides the Quantum Development Kit (QDK) that replaces the LIQUi|> programming environment [100] with a new programming language, called Q#. The QDK offers a library specifically for ML in Q# (https://learn.microsoft.com/en-us/azure/quantum/user-guide/libraries/, accessed on 3 April 2023).

The QDK includes a back-end circuit simulator and front-end support for the Q# language, integrated with Microsoft Visual Studio.

### 3.5. Nvidia CUDA Quantum

Nvidia, one of the leading GPU producers, recently developed a unified programming model called CUDA Quantum, designed explicitly for running heterogeneous workloads—as the one for PQC—with CPUs, GPUs, and QPUs working side by side (https://developer.nvidia.com/cuda-quantum, accessed on 3 April 2023). CUDA Quantum intends to support quantum hardware backends from different quantum computer partners, including Rigetti, Xanadu, and Pasqal to name a few. CUDA Quantum provides a C++-based programming model, and it is specifically designed to enable interoperable workflows with existing classical parallel programming models and compiler toolchains, such as Nvidia CUDA. Regarding quantum simulation technologies, Nvidia provides the cuQuantum Appliance and the cuQuantum SDK to accelerate HPC simulators with Nvidia GPUs.

Early experiments with CUDA Quantum include the development of benchmarking a GPU-accelerated hybrid QGAN [101] with a quantum generator and a classical discriminator [102].

### 3.6. OriginQ QPanda

QPanda is a software stack developed by the Chinese Origin Quantum that has launched a 6-Qubit and 2-Qubit superconducting quantum chip accessible via the cloud. QPanda provides both C++ and Python interfaces. Regarding PQC development, QPanda exploits the quantum machine learning VQNet library [103,104]. QPanda also provides several noiseless and adjustable simulation backends.

### 3.7. PennyLane

PennyLane is a Python library designed explicitly for differentiable computing, focusing on QNNs and quantum simulations. PennyLane is developed by Xanadu and is one of the best existing tools for prototyping and designing new QNN methods and architectures. The PennyLane framework can be divided into the following software components:

- **Pennylane Templates.** The software component provides higher-level building blocks for constructing QNNs. Templates are a library of ready-to-use templates of widely used PQC architectures. For instance, templates can be used to encode data into quantum states or to select pre-made QNN layers.
- **Gradients and Training.** This software layer provides optimization tools to train the quantum circuits. It includes automatic differentiation libraries, such as libraries from NumPy [105], PyTorch [21], JAX [106], and TensorFlow [20], and integrates them into the quantum computing framework.
- **Quantum Operators and Measurements.** This software layer provides different quantum operators, including quantum gates, noisy channels, state preparations, and measurements. As for the measurement, PennyLane supports results from quantum devices: observable expectation, its variance, single measurement samples, and computational basis state probabilities.
- **Quantum Circuit/Device** The software component provides the interface between the software and the hardware. In PennyLane, calculations involving the execution of one or more quantum circuits are formulated as quantum node objects. The quantum nodes are used to express the quantum circuit, pin the computation to a specific device, and execute it. This software layer comprises PennyLane **plugins** for different quantum hardware devices and simulators. These plugins enable users to execute quantum circuits on different devices and return the measurement outcomes.

PennyLane provides several quantum computer simulators, including a state simulator of qubit-based quantum systems, Gaussian states (for operations on CV architectures), qubit-based quantum circuit architectures written in TensorFlow for automatic differentiation, and qubit-based quantum circuit architectures for automatic differentiation with the autograd library [107].

### 3.8. Qiskit Machine Learning

The IBM Qiskit programming framework is one of the most popular and established approaches for programming quantum computers, as the IBM quantum systems were among the first to become available to the general public on the cloud. Qiskit provides an API to connect to and run a quantum code on the IBM quantum computers and a range of abstractions for gate-based quantum computing. Most importantly, for PQC and QNN development, Qiskit provides a library called qiskit-machine-learning, specifically designed to develop QNNs. At a high level, the qiskit-machine-learning framework can be divided into different software components:

- **Data Preparation.** This component is responsible for preprocessing the input data before it is used to train or test a quantum machine learning model.
- **Feature Maps.** The feature maps layer defines the quantum circuits that map the input data onto a quantum state. It includes pre-built feature maps for common ML tasks.
- **Neural Networks.** This component contains a programming interface for the QNNs (called NeuralNetwork) and two specific implementations (i) EstimatorQNN: this network is based on evaluating quantum mechanical observables, and (ii) SamplerQNN: a network based on the samples measuring a quantum circuit. These high-level classes provide methods for configuring the PQC, its initialization, and performing the forward and backward passes.
- **Classifiers and Regressors.** To train and use Quantum Neural Networks, qiskitmachine-learning provides different learning algorithms such as the NeuralNetworkClassifier and NeuralNetworkRegressor. These take a QNN as input and then use it for classification or regression. Two convenience implementations are provided to allow an easy start: the Variational Quantum Classifier (VQC) and the Variational Quantum Regressor (VQR).
- **Qiskit.** At the bottom of the qiskit-machine-learning software stack, there is Qiskit that provides quantum gate and circuits primitives (including parametrized gates), gradients, and optimizers.

In addition, qiskit-machine-learning provides a connector to PyTorch for implementing hybrid classical-quantum NNs, e.g., some nodes are classical, and some are quantum. This hybrid architecture is obtained by embedding a quantum layer in a classical PyTorch network. Regarding quantum computer simulators, the Qiskit Aer module provides different quantum computer simulator backends, including ideal and noisy state vectors, density matrix, and unitary simulation backends.

### 3.9. Rigetti Grove

The Rigetti Forest programming environment includes a quantum instruction language Quil, its Python interface, called pyQuil, and a library of quantum programs called Grove. Rigetti Grove is a collection of high-level primitives that can be used to develop QNNs. The Rigetti Forest also provides a quantum simulation environment called QVM (Quantum Virtual Machine).

### 3.10. Strawberry Fields

Strawberry Fields is a Python library designed to run quantum CV programs on quantum photonics hardware [108]. It is based on the language named Blackbird, and provides three different simulator backends: a simulator of Gaussian states, Fock states, and a Fock-basis backend written using the TensorFlow (that can provide automatic differentiation and optimizers). Regarding the PQC development, the TensorFlow backend is critical for optimizers and gradients from TensorFlow 2. Thanks to Strawberry Fields, it is possible to experiment and design a CV Quantum Neural Network, as discussed in the seminal paper on CV QNN [30].

### 3.11. TensorFlow Quantum

TensorFlow Quantum (TQ) is a Python library designed for ML workloads using quantum-classical QNN models [33]. TQ is developed by Google and leverages and unifies Google’s Cirq within TensorFlow. While integrating quantum computing algorithms and gates designed in Cirq, TQ delivers additional quantum computing primitives in line with the TensorFlow API and high-performance quantum circuit simulators. The basic TQ software layers are:

- **Classical and Quantum Data**. TFQ allows the processing of classical and quantum data (in the form of quantum circuits and operators).
- **Keras API**. TQ integrates with the core TensorFlow and Keras [109], providing NN models and optimizers.
- **Quantum Layers and Differentiators**. This part of the software stack provides hybrid quantum-classical automatic differentiation in connection with classical TensorFlow layers.
- **TensorFlow Ops**. This software component instantiates the dataflow graph, and custom operations regulate the quantum circuit execution.

In addition to Cirq, TQ also provides a high-performance C++ TQ-native (e.g., not relying on the Cirq simulators) quantum computer simulator for QNN called qsim.

### 3.12. Torch Quantum

Torch Quantum [67] is a PyTorch library designed explicitly for quantum machine learning and simulations at MIT. Torch Quantum leverages the main characteristics that made PyTorch popular and widespread in the data-science community: easy NN/PQC construction, dynamic computation graph for easier debugging, and gradient calculations via autograd. Torch Quantum can be easily deployed on real quantum devices such as IBM Quantum systems. Torch Quantum provides an HPC state vector simulator (with support for GPUs), and pulse simulation is planned to be implemented in the future.

### 3.13. Zapata Orquestra

Zapata offers a quantum computational platform, Orquestra, including a quantum SDK (for circuit, gate, and noise models) and an algorithm suite that comprises quantum ML, chemistry, cryptography, and error mitigation methods. Zapata developed a proprietary generative AI technique that exploits hybrid classical-quantum systems [110] and uses Quantum Circuit Born Machine (QCBM). Among the most important Orquestra features, there are the workflow manager and integration with deployment orchestration tools, such as Slurm and Ray, that allow for quantum-enabled workflows and execution on quantum and classical HPC resources. Orquestra supports different quantum computer backends, including IBM, D-Wave, IonQ systems, and the Qulacs quantum computer simulator.

### 3.14. Summary

To summarize the feature of the different QNN programming frameworks, we provide an overview of current QNN programming frameworks in Table 1, providing the target quantum architectures (possibly, also of future implementations), main programming languages, availability of quantum simulators, and distinctive features of the programming frameworks.

## 4. Conclusions

In this paper, we surveyed the current state-of-the-art high-level programming approaches for QNN development. We discussed target architectures, quantum data, critical QNN algorithmic components, such as the hybrid workflow of QA and PQC, optimizers, and techniques for performing gradient calculations on quantum computer hardware and simulators. We also presented existing programming QNN frameworks. The field of QNN methods and programming frameworks quickly evolves, and new techniques and methods will certainly develop to tackle current QNN limitations. Currently, one of the main QNN challenges is to address the problem of *barren plateau* in the optimization landscape.

Additional quantum computer architectures will become available for QNN developers and users in the future. An example is the PsiQuantum’s photonics fusion-based quantum chip [112] or the Microsoft topological quantum computers [113]. Despite the potential Cambrian explosion of different quantum computer architectures, programming these new quantum systems will likely retain the existing quantum computing abstractions (gates, circuit, measurements, QNN layer, …) and reuse existing programming approaches to ensure portability across different platforms, an important issue already in the HPC field. An example of a portable quantum programming framework is PennyLane, which allows for developing specific plugins to support different and possibly new QPU devices.

Following the existing development of machine learning frameworks, such as TensorFlow, it is likely that in the future, QNN frameworks will rely more and more on domain-specific languages and compiler technologies to provide an Intermediate Representation (IR) that can be translated to different quantum hardware (and simulator) backends. Compiler toolchains, such as LLVM and MLIR [114,115,116], are already in use by the Intel Quantum SDK [98], and CUDA Quantum. These technologies might have a prominent role in the future of programming QNN on a quantum computer.

## Figures and Tables

**Table 1:** Overview of different QNN frameworks for programming QNN on NISQ systems.

QNN Framework	Website (accessed on 3 April 2023)	Main Target Architecture	Language	QC Simulators	Distinctive Features
Amazon Braket SDK	https://github.com/aws/amazon-braket-sdk-python	Support Several QC Systems	Braket SDK, Python	Braket local and on-demand HPC simulators	Support Several QC Systems.
D-Wave Ocean	https://github.com/dwavesystems/dwave-ocean-sdk	D-Wave QAs	Python	OpenJIJ	QA Platform for Restricted Boltzmann Machines and energy-based ML
Intel HQCL, [98]	https://github.com/IntelLabs/Hybrid-Quantum-Classical-Library	Intel Quantum Dot-based QC (simulators/hardware)	C++	Intel Quantum Simulator (IQS)	Integration of compiler technologies and runtime
MS’s QDK	https://github.com/microsoft/Quantum	Support Several QC Systems	Q#/Python	MS’s QDK Circuit Simulator	Support Several QC Systems
Nvidia CUDA Quantum, [102]	https://developer.nvidia.com/cuda-quantum	GPU/QPU	C++	cuQuantum Appliance	Unified programming for heterogeneous QPU, GPU, and CPU systems
OriginQ QPanda, [111]	https://github.com/OriginQ/QPanda-2	Origin Quantum QC	Python	Several Simulators	Integration with VQNet library for PQC
PennyLane, [78]	https://github.com/PennyLaneAI/pennylane	Photonics QC	Python	State simulator of qubit-based quantum systems, Gaussian states, TensorFlow and PyTorch autograd	Ideal for prototyping and designing new methods. Support for discrete and CV QC
Qiskit-machine-learning	https://github.com/Qiskit/qiskit-machine-learning	IBM QC	Python	Qiskit Aer	QNN, Estimator, and Sampler Abstractions. Integration with PyTorch
Rigetti Grove	https://github.com/rigetti/grove	Rigetti Quantum Computers	PiQuil/Python	QVM (Quantum Virtual Machine)	Full software stack
Strawberry Fields, [108]	https://github.com/XanaduAI/strawberryfields	CV Quantum Computing, Photonic CV	Blackbird/Python	Simulator with Gaussian states and Fock states.	Integration with TensorFlow 2 as backend: TF optimizers and automatic differentiation.
TensorFlow Quantum, [33]	https://github.com/tensorflow/quantum	Gate-based Google QC	Integration with Keras, Tensorflow, Python	qsim	Tight integration with TensorFlow, Keras, and Cirq
Torch Quantum, [67]	https://github.com/mit-han-lab/torchquantum	IBM	Python	Simulator Backend, Planned pulse simulator	Easy PQC construction, dynamic computation graph, gradient calculations via autograd
Zapata Orquestra, [110]	https://github.com/zapatacomputing	IBM, D-Wave, Rigetti, IonQ	Python	Qulacs	Quantum-enabled workflows

## Data Availability

Not applicable.

## Abbreviations

The following abbreviations are used in this manuscript:

AWS	Amazon Web Services
CV	Continuous Variable
DBN	Deep Belief Network
DQC	Differentiable Quantum Circuit
GPU	Graphical Processing Unit
HHL	Harrow–Hassidim–Lloyd
HPC	High-Performance Computing
ML	Machine Learning
NISQ	Noisy Intermediate-Scale Quantum
NN	Neural Network
PCA	Principal Component Analysis
PINN	Physics-Informed Neural Network
PQC	Parametrized Quantum Circuit
QA	Quantum Annealer
qBLAS	Quantum Basic Linear Algebra Subprograms
QCBM	Quantum Circuit Born Machine
QFT	Quantum Fourier Transform
QML	Quantum Machine Learning
QNN	Quantum Neural Network
QPE	Quantum Phase Estimation
QUBO	Quadratic Unconstrained Binary Optimization
RBM	Restricted Boltzmann Machine
SDK	Software Development Toolkit
SPSA	Simultaneous Perturbation Stochastic Approximation
SVD	Singular Value Decomposition
TQ	TensorFlow Quantum
VQC	Variational Quantum Circuits

## References

[1] Nielsen M.A., Chuang I. (2002). Quantum Computation and Quantum Information.

[2] Rieffel E.G., Polak W.H. (2011). Quantum Computing: A Gentle Introduction.

[3] Bravyi S., Gosset D., König R. (2018). Quantum advantage with shallow circuits. Science.

[4] Biamonte J., Wittek P., Pancotti N., Rebentrost P., Wiebe N., Lloyd S. (2017). Quantum machine learning. Nature.

[5] Schuld M., Petruccione F. (2018). Supervised Learning with Quantum Computers.

[6] Harrow A.W., Hassidim A., Lloyd S. (2009). Quantum algorithm for linear systems of equations. Phys. Rev. Lett..

[7] Lloyd S., Mohseni M., Rebentrost P. (2014). Quantum principal component analysis. Nat. Phys..

[8] Wiebe N., Braun D., Lloyd S. (2012). Quantum algorithm for data fitting. Phys. Rev. Lett..

[9] Lloyd S., Garnerone S., Zanardi P. (2016). Quantum algorithms for topological and geometric analysis of data. Nat. Commun..

[10] Low G.H., Yoder T.J., Chuang I.L. (2014). Quantum inference on Bayesian networks. Phys. Rev. A.

[11] Rebentrost P., Mohseni M., Lloyd S. (2014). Quantum support vector machine for big data classification. Phys. Rev. Lett..

[12] Giovannetti V., Lloyd S., Maccone L. (2008). Quantum random access memory. Phys. Rev. Lett..

[13] Preskill J. (2018). Quantum computing in the NISQ era and beyond. Quantum.

[14] Schuld M., Killoran N. (2022). Is quantum advantage the right goal for quantum machine learning?. Prx Quantum.

[15] Boixo S., Smelyanskiy V.N., Shabani A., Isakov S.V., Dykman M., Denchev V.S., Amin M.H., Smirnov A.Y., Mohseni M., Neven H. (2016). Computational multiqubit tunnelling in programmable quantum annealers. Nat. Commun..

[16] Heim B., Soeken M., Marshall S., Granade C., Roetteler M., Geller A., Troyer M., Svore K. (2020). Quantum programming languages. Nat. Rev. Phys..

[17] Cross A., Javadi A., Alexander T., Bishop L., Ryan C.A., Heidel S., de Beaudrap N., Smolin J., Gambetta J., Johnson B.R. Open Quantum Assembly Language. Proceedings of the ACM SIGPLAN Conference on Programming Language Design and Implementation.

[18] Wille R., Van Meter R., Naveh Y. IBM’s Qiskit tool chain: Working with and developing for real quantum computers. Proceedings of the 2019 Design, Automation & Test in Europe Conference & Exhibition (2019).

[19] Smith R.S., Curtis M.J., Zeng W.J. (2016). A practical quantum instruction set architecture. arXiv.

[20] Abadi M., Barham P., Chen J., Chen Z., Davis A., Dean J., Devin M., Ghemawat S., Irving G., Isard M. Tensorflow: A system for large-scale machine learning. Proceedings of the Osdi.

[21] Paszke A., Gross S., Massa F., Lerer A., Bradbury J., Chanan G., Killeen T., Lin Z., Gimelshein N., Antiga L. Pytorch: An imperative style, high-performance deep learning library. Proceedings of the 33rd International Conference on Neural Information Processing Systems.

[22] Yarkoni S., Raponi E., Bäck T., Schmitt S. (2022). Quantum annealing for industry applications: Introduction and review. Rep. Prog. Phys..

[23] Aramon M., Rosenberg G., Valiante E., Miyazawa T., Tamura H., Katzgraber H.G. (2019). Physics-inspired optimization for quadratic unconstrained problems using a digital annealer. Front. Phys..

[24] Nakayama H., Koyama J., Yoneoka N., Miyazawa T. (2021). Description: Third Generation Digital Annealer Technology.

[25] Goto H. (2019). Quantum computation based on quantum adiabatic bifurcations of Kerr-nonlinear parametric oscillators. J. Phys. Soc. Jpn..

[26] Susa Y., Nishimori H. (2021). Variational optimization of the quantum annealing schedule for the Lechner-Hauke-Zoller scheme. Phys. Rev. A.

[27] Kaye P., Laflamme R., Mosca M. (2006). An Introduction to Quantum Computing.

[28] Henriet L., Beguin L., Signoles A., Lahaye T., Browaeys A., Reymond G.O., Jurczak C. (2020). Quantum computing with neutral atoms. Quantum.

[29] Lloyd S., Braunstein S.L. (1999). Quantum computation over continuous variables. Phys. Rev. Lett..

[30] Killoran N., Bromley T.R., Arrazola J.M., Schuld M., Quesada N., Lloyd S. (2019). Continuous-variable quantum neural networks. Phys. Rev. Res..

[31] Markidis S. (2022). On the Physics-Informed Neural Networks for Quantum Computers. arXiv.

[32] LaRose R., Coyle B. (2020). Robust data encodings for quantum classifiers. Phys. Rev. A.

[33] Broughton M., Verdon G., McCourt T., Martinez A.J., Yoo J.H., Isakov S.V., Massey P., Halavati R., Niu M.Y., Zlokapa A. (2020). Tensorflow quantum: A software framework for quantum machine learning. arXiv.

[34] McClean J.R., Rubin N.C., Sung K.J., Kivlichan I.D., Bonet-Monroig X., Cao Y., Dai C., Fried E.S., Gidney C., Gimby B. (2020). OpenFermion: The electronic structure package for quantum computers. Quantum Sci. Technol..

[35] Sun Q., Berkelbach T.C., Blunt N.S., Booth G.H., Guo S., Li Z., Liu J., McClain J.D., Sayfutyarova E.R., Sharma S. (2018). PySCF: The Python-based simulations of chemistry framework. Wiley Interdiscip. Rev. Comput. Mol. Sci..

[36] Hu F., Wang B.N., Wang N., Wang C. (2019). Quantum machine learning with D-wave quantum computer. Quantum Eng..

[37] Nath R.K., Thapliyal H., Humble T.S. (2021). A review of machine learning classification using quantum annealing for real-world applications. SN Comput. Sci..

[38] Boothby T., King A.D., Roy A. (2016). Fast clique minor generation in Chimera qubit connectivity graphs. Quantum Inf. Process..

[39] Klymko C., Sullivan B.D., Humble T.S. (2014). Adiabatic quantum programming: Minor embedding with hard faults. Quantum Inf. Process..

[40] MacKay D.J., Mac Kay D.J. (2003). Information Theory, Inference and Learning Algorithms.

[41] Bauckhage C., Sanchez R., Sifa R. Problem solving with Hopfield networks and adiabatic quantum computing. Proceedings of the 2020 International Joint Conference on Neural Networks (IJCNN).

[42] Dorband J.E. A Boltzmann machine implementation for the d-wave. Proceedings of the 2015 12th International Conference on Information Technology-New Generations.

[43] Dixit V., Selvarajan R., Alam M.A., Humble T.S., Kais S. (2021). Training restricted boltzmann machines with a d-wave quantum annealer. Front. Phys..

[44] Adachi S.H., Henderson M.P. (2015). Application of quantum annealing to training of deep neural networks. arXiv.

[45] Benedetti M., Lloyd E., Sack S., Fiorentini M. (2019). Parameterized quantum circuits as machine learning models. Quantum Sci. Technol..

[46] Farhi E., Neven H. (2018). Classification with quantum neural networks on near term processors. arXiv.

[47] Chen H., Wossnig L., Severini S., Neven H., Mohseni M. (2021). Universal discriminative quantum neural networks. Quantum Mach. Intell..

[48] Ruder S. (2016). An overview of gradient descent optimization algorithms. arXiv.

[49] Kingma D.P., Ba J. (2014). Adam: A method for stochastic optimization. arXiv.

[50] Cong I., Choi S., Lukin M.D. (2019). Quantum convolutional neural networks. Nat. Phys..

[51] Henderson M., Shakya S., Pradhan S., Cook T. (2020). Quanvolutional neural networks: Powering image recognition with quantum circuits. Quantum Mach. Intell..

[52] Huang H.L., Du Y., Gong M., Zhao Y., Wu Y., Wang C., Li S., Liang F., Lin J., Xu Y. (2021). Experimental quantum generative adversarial networks for image generation. Phys. Rev. Appl..

[53] Huggins W., Patil P., Mitchell B., Whaley K.B., Stoudenmire E.M. (2019). Towards quantum machine learning with tensor networks. Quantum Sci. Technol..

[54] McClean J.R., Boixo S., Smelyanskiy V.N., Babbush R., Neven H. (2018). Barren plateaus in quantum neural network training landscapes. Nat. Commun..

[55] Grant E., Wossnig L., Ostaszewski M., Benedetti M. (2019). An initialization strategy for addressing barren plateaus in parametrized quantum circuits. Quantum.

[56] Cerezo M., Sone A., Volkoff T., Cincio L., Coles P.J. (2021). Cost function dependent barren plateaus in shallow parametrized quantum circuits. Nat. Commun..

[57] Pérez-Salinas A., Cervera-Lierta A., Gil-Fuster E., Latorre J.I. (2020). Data re-uploading for a universal quantum classifier. Quantum.

[58] Wolf M.M. (2012). Quantum Channels & Operations: Guided Tour.

[59] Schuld M., Killoran N. (2019). Quantum machine learning in feature Hilbert spaces. Phys. Rev. Lett..

[60] Schuld M., Sweke R., Meyer J.J. (2021). Effect of data encoding on the expressive power of variational quantum-machine-learning models. Phys. Rev. A.

[61] Havlíček V., Córcoles A.D., Temme K., Harrow A.W., Kandala A., Chow J.M., Gambetta J.M. (2019). Supervised learning with quantum-enhanced feature spaces. Nature.

[62] Kyriienko O., Paine A.E., Elfving V.E. (2021). Solving nonlinear differential equations with differentiable quantum circuits. Phys. Rev. A.

[63] Paine A.E., Elfving V.E., Kyriienko O. (2022). Quantum kernel methods for solving differential equations. arXiv.

[64] Heim N., Ghosh A., Kyriienko O., Elfving V.E. (2021). Quantum model-discovery. arXiv.

[65] Schuld M., Bocharov A., Svore K.M., Wiebe N. (2020). Circuit-centric quantum classifiers. Phys. Rev. A.

[66] Chen G., Chen Q., Long S., Zhu W., Yuan Z., Wu Y. (2022). Quantum convolutional neural network for image classification. Pattern Anal. Appl..

[67] Wang H., Ding Y., Gu J., Lin Y., Pan D.Z., Chong F.T., Han S. QuantumNAS: Noise-adaptive search for robust quantum circuits. Proceedings of the 2022 IEEE International Symposium on High-Performance Computer Architecture (HPCA).

[68] Du Y., Huang T., You S., Hsieh M.H., Tao D. (2022). Quantum circuit architecture search for variational quantum algorithms. NPJ Quantum Inf..

[69] Zhu D., Linke N.M., Benedetti M., Landsman K.A., Nguyen N.H., Alderete C.H., Perdomo-Ortiz A., Korda N., Garfoot A., Brecque C. (2019). Training of quantum circuits on a hybrid quantum computer. Sci. Adv..

[70] Nelder J.A., Mead R. (1965). A simplex method for function minimization. Comput. J..

[71] Bonet-Monroig X., Wang H., Vermetten D., Senjean B., Moussa C., Bäck T., Dunjko V., O’Brien T.E. (2021). Performance comparison of optimization methods on variational quantum algorithms. arXiv.

[72] Virtanen P., Gommers R., Oliphant T.E., Haberland M., Reddy T., Cournapeau D., Burovski E., Peterson P., Weckesser W., Bright J. (2020). SciPy 1.0: Fundamental algorithms for scientific computing in Python. Nat. Methods.

[73] Sweke R., Wilde F., Meyer J., Schuld M., Fährmann P.K., Meynard-Piganeau B., Eisert J. (2020). Stochastic gradient descent for hybrid quantum-classical optimization. Quantum.

[74] Spall J.C. (1998). An overview of the simultaneous perturbation method for efficient optimization. Johns Hopkins Apl Tech. Dig..

[75] Stokes J., Izaac J., Killoran N., Carleo G. (2020). Quantum natural gradient. Quantum.

[76] Amari S.I. (1998). Natural gradient works efficiently in learning. Neural Comput..

[77] Baydin A.G., Pearlmutter B.A., Radul A.A., Siskind J.M. (2018). Automatic differentiation in machine learning: A survey. J. Marchine Learn. Res..

[78] Bergholm V., Izaac J., Schuld M., Gogolin C., Alam M.S., Ahmed S., Arrazola J.M., Blank C., Delgado A., Jahangiri S. (2018). Pennylane: Automatic differentiation of hybrid quantum-classical computations. arXiv.

[79] Guerreschi G.G., Smelyanskiy M. (2017). Practical optimization for hybrid quantum-classical algorithms. arXiv.

[80] Schuld M., Bergholm V., Gogolin C., Izaac J., Killoran N. (2019). Evaluating analytic gradients on quantum hardware. Phys. Rev. A.

[81] Wierichs D., Izaac J., Wang C., Lin C.Y.Y. (2022). General parameter-shift rules for quantum gradients. Quantum.

[82] Jones T., Gacon J. (2020). Efficient calculation of gradients in classical simulations of variational quantum algorithms. arXiv.

[83] Koczor B., Benjamin S.C. (2022). Quantum analytic descent. Phys. Rev. Res..

[84] Liu J., Spedalieri F.M., Yao K.T., Potok T.E., Schuman C., Young S., Patton R., Rose G.S., Chamka G. (2018). Adiabatic quantum computation applied to deep learning networks. Entropy.

[85] Li R.Y., Di Felice R., Rohs R., Lidar D.A. (2018). Quantum annealing versus classical machine learning applied to a simplified computational biology problem. NPJ Quantum Inf..

[86] Mott A., Job J., Vlimant J.R., Lidar D., Spiropulu M. (2017). Solving a Higgs optimization problem with quantum annealing for machine learning. Nature.

[87] Konar D., Gelenbe E., Bhandary S., Sarma A.D., Cangi A. (2022). Random quantum neural networks (RQNN) for noisy image recognition. arXiv.

[88] Suryotrisongko H., Musashi Y. (2022). Evaluating hybrid quantum-classical deep learning for cybersecurity botnet DGA detection. Procedia Comput. Sci..

[89] Shahwar T., Zafar J., Almogren A., Zafar H., Rehman A.U., Shafiq M., Hamam H. (2022). Automated detection of Alzheimer’s via hybrid classical quantum neural networks. Electronics.

[90] Blance A., Spannowsky M. (2021). Quantum machine learning for particle physics using a variational quantum classifier. J. High Energy Phys..

[91] Guan W., Perdue G., Pesah A., Schuld M., Terashi K., Vallecorsa S., Vlimant J.R. (2021). Quantum machine learning in high energy physics. Mach. Learn. Sci. Technol..

[92] Otgonbaatar S., Datcu M. (2021). Classification of remote sensing images with parameterized quantum gates. IEEE Geosci. Remote Sens. Lett..

[93] Sengupta K., Srivastava P.R. (2021). Quantum algorithm for quicker clinical prognostic analysis: An application and experimental study using CT scan images of COVID-19 patients. BMC Med. Inform. Decis. Mak..

[94] Garcia-Alonso J., Rojo J., Valencia D., Moguel E., Berrocal J., Murillo J.M. (2021). Quantum software as a service through a quantum API gateway. IEEE Internet Comput..

[95] Liu D.C., Nocedal J. (1989). On the limited memory BFGS method for large scale optimization. Math. Program..

[96] Zaman M., Tanahashi K., Tanaka S. (2021). PyQUBO: Python library for mapping combinatorial optimization problems to QUBO form. IEEE Trans. Comput..

[97] Wu X.C., Khalate P., Schmitz A., Premaratne S., Rasch K., Daraeizadeh S., Kotlyar R., Ren S., Paykin J., Rose F. (2023). Intel Quantum SDK Version 1.0: Extended C++ Compiler, Runtime and Quantum Hardware Simulators for Hybrid Quantum-Classical Applications. Bull. Am. Phys. Soc..

[98] Khalate P., Wu X.C., Premaratne S., Hogaboam J., Holmes A., Schmitz A., Guerreschi G.G., Zou X., Matsuura A. (2022). An LLVM-based C++ Compiler Toolchain for Variational Hybrid Quantum-Classical Algorithms and Quantum Accelerators. arXiv.

[99] Matsuura A., Premaratne S., Wu X.C., Sawaya N., Schmitz A., Khalate P., Daraeizadeh S., Guerreschi G.G., Khammassi N., Rasch K. An Intel Quantum Software Development Kit for Efficient Execution of Variational Algorithms. Proceedings of the APS March Meeting Abstracts.

[100] Wecker D., Svore K.M. (2014). LIQUi|>: A software design architecture and domain-specific language for quantum computing. arXiv.

[101] Ngo T.A., Nguyen T., Thang T.C. (2023). A Survey of Recent Advances in Quantum Generative Adversarial Networks. Electronics.

[102] Rao P., Chandani Z., Wilson A., Schweitz E., Schmitt B., Santana A., Lelbach B., McCaskey A. (2023). Benchmarking of quantum generative adversarial networks using NVIDIA’s Quantum Optimized Device Architecture. Bull. Am. Phys. Soc..

[103] Chen Z.Y., Xue C., Chen S.M., Guo G.P. (2019). Vqnet: Library for a quantum-classical hybrid neural network. arXiv.

[104] Bian H., Jia Z., Dou M., Fang Y., Li L., Zhao Y., Wang H., Zhou Z., Wang W., Zhu W. (2023). VQNet 2.0: A New Generation Machine Learning Framework that Unifies Classical and Quantum. arXiv.

[105] Van Der Walt S., Colbert S.C., Varoquaux G. (2011). The NumPy array: A structure for efficient numerical computation. Comput. Sci. Eng..

[106] Frostig R., Johnson M.J., Leary C. (2018). Compiling machine learning programs via high-level tracing. Syst. Mach. Learn..

[107] Paszke A., Gross S., Chintala S., Chanan G., Yang E., DeVito Z., Lin Z., Desmaison A., Antiga L., Lerer A. (2017). Automatic differentiation in pytorch. https://openreview.net/forum?id=BJJsrmfCZ.

[108] Killoran N., Izaac J., Quesada N., Bergholm V., Amy M., Weedbrook C. (2019). Strawberry fields: A software platform for photonic quantum computing. Quantum.

[109] Gulli A., Pal S. (2017). Deep Learning with Keras.

[110] Hibat-Allah M., Mauri M., Carrasquilla J., Perdomo-Ortiz A. (2023). A Framework for Demonstrating Practical Quantum Advantage: Racing Quantum against Classical Generative Models. arXiv.

[111] Dou M., Zou T., Fang Y., Wang J., Zhao D., Yu L., Chen B., Guo W., Li Y., Chen Z. (2022). QPanda: High-performance quantum computing framework for multiple application scenarios. arXiv.

[112] Bartolucci S., Birchall P., Bombin H., Cable H., Dawson C., Gimeno-Segovia M., Johnston E., Kieling K., Nickerson N., Pant M. (2023). Fusion-based quantum computation. Nat. Commun..

[113] Nayak C., Simon S.H., Stern A., Freedman M., Sarma S.D. (2008). Non-Abelian anyons and topological quantum computation. Rev. Mod. Phys..

[114] McCaskey A., Nguyen T. A MLIR dialect for quantum assembly languages. Proceedings of the 2021 IEEE International Conference on Quantum Computing and Engineering (QCE).

[115] Ittah D., Häner T., Kliuchnikov V., Hoefler T. (2022). QIRO: A static single assignment-based quantum program representation for optimization. ACM Trans. Quantum Comput..

[116] Ittah D., Häner T., Kliuchnikov V., Hoefler T. (2021). Enabling dataflow optimization for quantum programs. arXiv.

